# Optimization and scalability assessment of supercapacitor electrodes based on hydrothermally grown MoS_2_ on carbon cloth†

**DOI:** 10.1039/d4na00368c

**Published:** 2024-07-18

**Authors:** Jasna Mannayil, Olli Pitkänen, Minna Mannerkorpi, Krisztian Kordas

**Affiliations:** a Microelectronics Research Unit, University of Oulu Erkki Koiso-Kanttilan katu 3 90570 Oulu Finland; b Research Unit of Health Sciences and Technology, University of Oulu 90220 Oulu Finland jasna.mannayil@oulu.fi olli.pitkanen@oulu.fi

## Abstract

MoS_2_ is a well-known 2D transition metal dichalcogenide (TMD) with feasibility for energy storage applications due to its eco-friendliness and high electroactive surface area. Electrodes based on MoS_2_ are typically made by either immobilizing its multiphase nanocomposites, having binders and conductive fillers, or by directly growing the materials on current collectors. In this work, we follow and optimize this latter approach by applying a hydrothermal route to directly synthesize MoS_2_ nanostructures on carbon cloth (MoS_2_@CC) hence enabling binder-free current collector electrodes. Raman spectroscopy and electron microscopy analyses confirmed the formation of 2H MoS_2_ nanosheets with hexagonal structure. The as-prepared electrodes were used to assemble symmetric supercapacitor cells, whose performance were tested in various types of electrolytes. Electrochemical measurements indicate that both precursor concentration and growth time significantly affect the device performance. Under optimized conditions, specific capacitance up to 226 F g^−1^ (at 1 A g^−1^ in 6 M KOH) was achieved, with corresponding energy and power densities of 5.1 W h kg^−1^ and 2.1 W kg^−1^. The device showed good stability, retaining 85% capacitance after 1000 cycles. Furthermore, the electrodes assessed in PYR14-TFSI showed energy and power densities of up to 26.3 W h kg^−1^ and 2.0 kW kg^−1^, respectively, indicating their feasibility not only in aqueous but also in ionic liquid electrolytes. In addition, galvanostatic charge/discharge measurements conducted on devices having footprint sizes from 1 cm^2^ to 25 cm^2^ show very similar specific capacitances, which proves scalability and thus the practical relevance of the binder-free electrodes demonstrated in this study.

## Abstract 2

Hydrothermal synthesis of binder-free MoS_2_ on carbon cloth is optimized and demonstrated using aqueous and ionic liquid-based electrolytes for application in supercapacitors.

## Graphical abstract

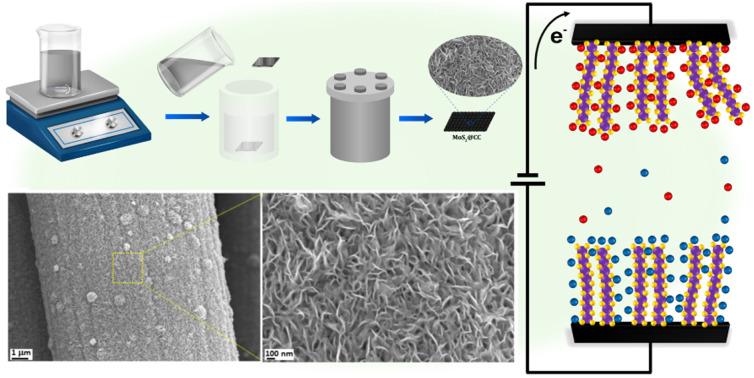

## 1. Introduction

Molybdenum disulfide (MoS_2_) is one of the most explored 2D materials after graphene. MoS_2_ exists in both metallic (1T trigonal) and semiconducting (2H hexagonal and 3R rhombohedral) forms, among which the 2H phase is the most stable. The enormous variety of the so far synthesized material structures, the relatively easy insertion of small ions into the interlayer spacing, the abundant defect sites at the edges of layers, the excellent in-plane carrier transport, and fascinating optoelectronic properties make MoS_2_ and its derivatives and composites particularly attractive for applications in catalysis and photocatalysis,^1,2^ chemical and electrochemical sensors,^3^ photodetectors,^4^ and transistors^5,6^ as well as batteries^7,8^ and capacitors.^9–13^

MoS_2_ can be synthesized by top-down methods such as chemical/mechanical exfoliation,^14–16^ and bottom-up techniques including chemical vapor deposition,^4,17,18^ atomic layer deposition,^19,20^ pulsed laser deposition (PLD),^21,22^ and RF-magnetron sputtering,^23–25^ as well as wet chemical routes under normal^26^ and hydrothermal conditions.^27,28^ Among these methods, hydrothermal growth is probably the most practical for scale-up synthesis with high yield.^8^

Supercapacitor electrodes are usually prepared using the conventional slurry method, which involves applying a paste (having a typical composition of 80% active material, 10% polymer binder, and 10% conductive filler) on the collectors by means of spray coating,^29^ doctor blading,^30^ and printing or painting.^31^ Recently, also more research has focused on electrode design to improve the electrode material energy storage performance.^32–34^ The use of inactive polymeric binders reduces the performance of supercapacitors. Therefore, methods that are suitable to directly grow or deposit the active materials on the collectors (*e.g.*, metal plates, porous metal foams, carbon cloths) without using binders and additives are favorable provided detachment/leaching of the active layer is avoided.^35–38^ Accordingly, the recent trend is to directly synthesize MoS_2_ on conductive current collectors to be used as electrodes.^39–42^

In this work, we report scalable synthesis of binder-free MoS_2_-based supercapacitor electrodes and their practicality in scaling up the device by directly synthesizing vertically aligned MoS_2_ nanosheets on highly conducting and flexible carbon cloth (CC) current collectors using a hydrothermal route. Symmetric supercapacitors based on the obtained MoS_2_@CC structures were assembled and their electrochemical performance was optimized by varying the precursor concentration and growth time of the MoS_2_ synthesis. The developed SCs showed a specific capacitance of up to 226 F g^−1^ with a retention of 85% after 1000 cycles, suggesting feasibility for high-power supercapacitor applications. To increase the voltage window, the developed electrodes were also tested in a solvent-free ionic liquid (IL), 1-butyl-1-methylpyrrolidinium bis(trifluoromethanesulfonyl)imide (PYR14-TFSI) as well as in PYR14-TFSI mixed in acetonitrile (ACN), and it was found that the electrode in the IL/ACN mixture showed good electrochemical performance with the highest measured energy and power density of 26.3 W h kg^−1^ and 2.0 kW kg^−1^, respectively. The practical feasibility of scaling-up the developed supercapacitor (SC) electrode was evaluated using sandwich-type SCs with varying electrode sizes ranging from 1 cm^2^ to 25 cm^2^. The specific capacitance of the scaled-up devices is consistent with that of the 1 cm^2^ device, affirming the developed electrode's practical utility in high-performance supercapacitors.

## 2. Experimental

### 2.1 Materials

Sodium molybdate dihydrate (Na_2_MoO_4_·2H_2_O) and thiourea (CH_4_N_2_S) were obtained from Sigma Aldrich. Plain carbon cloth (#1071, fiber diameter of 5–10 μm) was purchased from the Fuel Cell Store, USA. Other solvents of analytical grade were used without further purification.

### 2.2 Surface treatment of carbon cloth

Bare carbon cloth is hard to wet in an aqueous solution due to its hydrophobic nature. To obtain a hydrophilic surface and uniform growth of MoS_2_, carbon cloth was subjected to a surface treatment using a protocol similar to that of Zhang *et al.*^43^ Pieces of CC were cut to a size of 5 × 5 cm^2^, then cleaned with a mixture of ethanol and acetone (1 : 1 vol.) and rinsed in DI water. The cleaned CCs were immersed in a mixture of cc. H_2_SO_4_ : HNO_3_ (1 : 1 vol.) and subjected to an ultrasonic treatment for 60 minutes. Finally, the surface-treated carbon cloth was washed with DI water and then dried at 60 °C overnight.

### 2.3 Synthesis of MoS_2_ on carbon cloth

MoS_2_ nanosheets were grown on CC using a hydrothermal technique from sodium molybdate dihydrate (Na_2_MoO_4_·2H_2_O) and thiourea (CH_4_N_2_S) as molybdenum and sulfur sources, respectively.^39,41,44,45^ The precursors were dissolved in 600 mL DI water. The solution was magnetically stirred for 1 h and then transferred into a 750 mL Teflon-lined vessel in an autoclave. After the CC was immersed in the solution, the vessel was closed and heated to 200 °C. After synthesis, MoS_2_ coated carbon cloth (MoS_2_@CC) was washed with DI water followed by rinsing in ethanol and drying at 60 °C overnight. To investigate the effect of synthesis parameters, reaction time and precursor concentrations were varied, and samples were named based on molybdenum precursor concentrations as MC005 (0.005 M sodium molybdate dihydrate and 0.025 M thiourea), MC01 (0.01 M sodium molybdate dihydrate and 0.05 M thiourea), and MC02 (0.02 M sodium molybdate dihydrate and 0.1 M thiourea).

### 2.4 Materials characterization

The crystal structure of the grown MoS_2_ on CC was characterized by X-ray diffraction (XRD, Rigaku SmartLab 9 kW, Co source) and Raman spectroscopy (Thermo Fisher Scientific A DXR TM 2xi, *λ* = 532 nm), whereas the morphology and microstructure were assessed using field-emission scanning electron microscopy (FESEM, Zeiss Ultra Plus) and transmission electron microscopy (TEM, JEOL JEM-2200FS).

### 2.5 Electrochemical measurements

As-prepared MoS_2_@CC was punched into circular discs of 1 cm in diameter and then symmetric supercapacitor cells were assembled in a Swagelok cell together with a filter paper as the separator. Three different electrolytes were used in the experiments: (i) 6 M KOH (aq.), (ii) 1-butyl-1-methylpyrrolidinium bis(trifluoromethanesulfonyl)imide (PYR14-TFSI), and (iii) a mixture of PYR14-TFSI and acetonitrile (ACN) with 1 : 1 mass ratio. In scaling-up experiments, the supercapacitors were assembled between stainless steel sheets of varying sizes and clamped together. Electrochemical measurements including cyclic voltammetry (CV), galvanostatic charge/discharge (GCD), and electrochemical impedance spectroscopy (EIS) were carried out using a VersaSTAT 3 instrument. The specific capacitances of the SCs were calculated from the GCD curves as:1
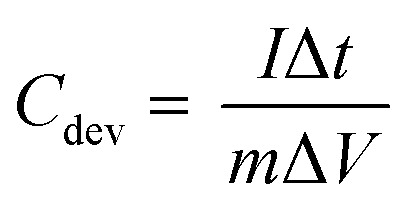
where *C*_dev_ is the device capacitance, *I* is the current, Δ*t* is the discharge time, *m* is the total mass of active materials, and Δ*V* is the voltage window. Note: the specific capacitance of a single electrode is calculated as *C*_elec_ = 4*C*_dev_. The energy density (*E* in W h kg^−1^) and power density (*P* in W kg^−1^) of the SC are calculated using eqn (2) and (3), respectively.^40–42^2
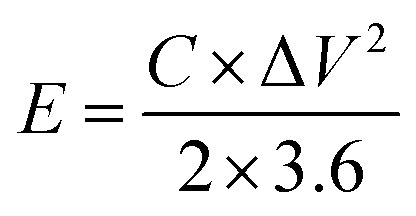
3
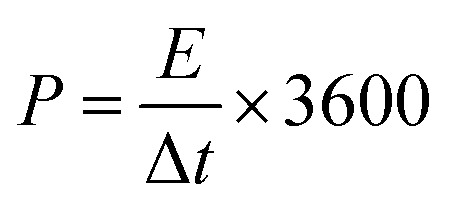
where Δ*t* is the discharge time in seconds.

## 3. Results and discussion

**Fig. 1:**
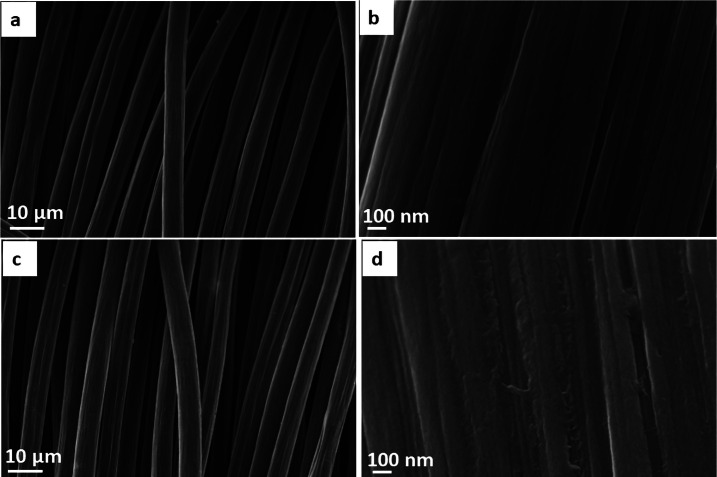
FESEM images of (a and b) bare CC and (c and d) acid-treated CC.

The three-dimensional fiber-like structured CC acts as a template for the growth of MoS_2_ nanosheets. Surface-treated CC was characterized with FESEM to analyze the surface morphology of carbon fibers. The surface of bare CC was very smooth, as shown in Fig. 1a, while some cracks were observed in acid-treated CC (Fig. 1c). In addition, as suggested by multiple studies for carbon fibers^43,46^ and nanotubes,^47^ treatment with oxidizing acids results in the partial etching/cutting of the carbonaceous structure^48^ as well as in the formation of polar functional groups (such as –COOH, –OH, –C=O) turning the originally hydrophobic character of the materials to hydrophilic. Accordingly, the acid treatment helps to improve the wettability of the surface by the precursor solution and allows for uniform nucleation and growth of subsequently anchored nanoparticles^49^ – MoS_2_ nanosheets in our study.

Hydrothermal reaction of a precursor solution containing MoO_4_^2−^ and SO_4_^2−^ leads to the formation of MoS_2_ nanosheets. During the process, CH_4_N_2_S decomposes partly into H_2_S, whereas Na_2_MoO_4_·2H_2_O into MoO_3_ and reacts to eventually form MoS_2_ (eqn (4)–(6)):^50^

4 CH_4_N_2_S + 2H_2_O → 2NH_3_ + CO_2_ + H_2_S

5 Na_2_MoO_4_·2H_2_O + H_2_O → MoO_3_ + 2NaOH + 2H_2_O

6 9H_2_S + 4MoO_3_ + 2NaOH → 4MoS_2_ + Na_2_SO_4_ + 10H_2_O

**Fig. 2:**
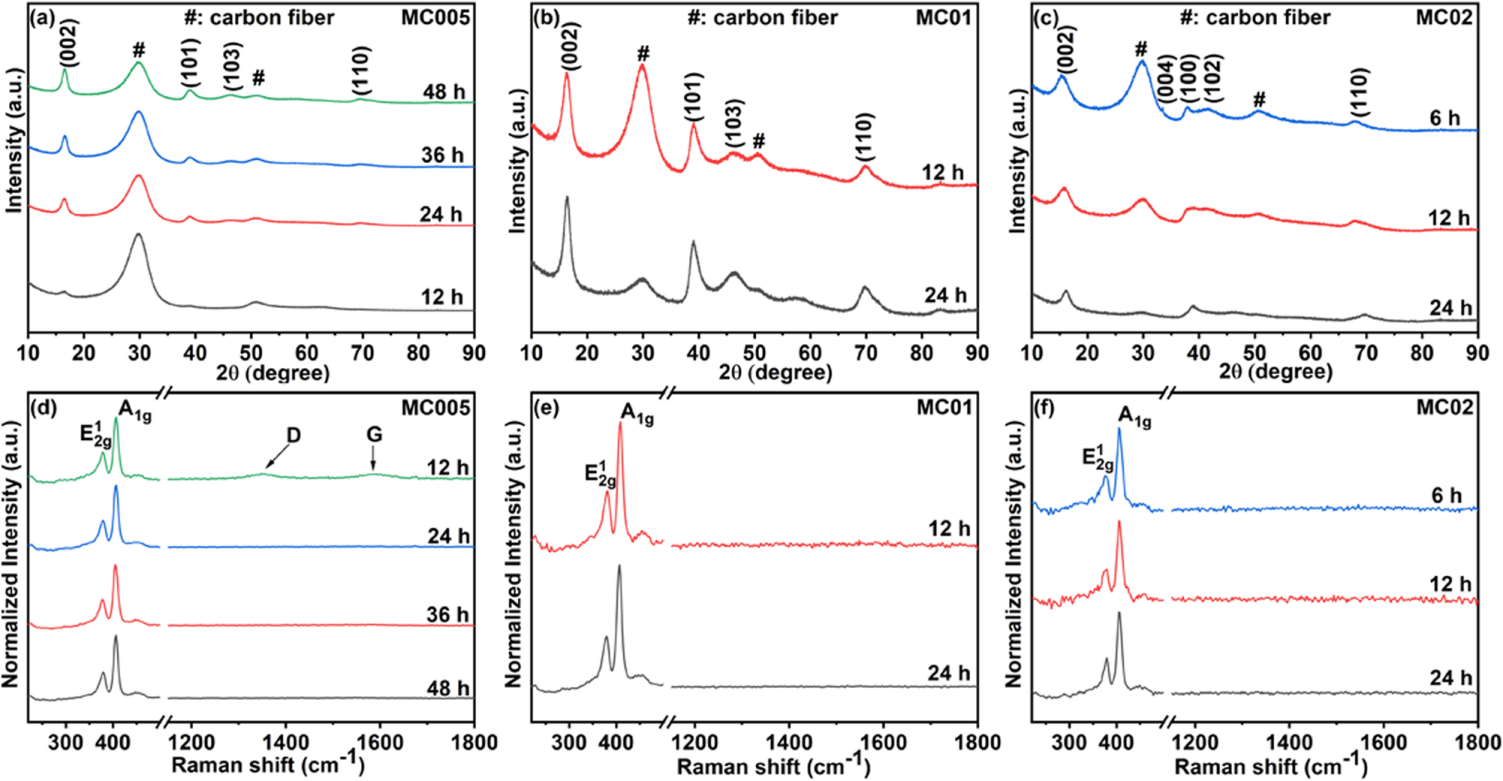
(a–c) XRD patterns and (d–f) Raman spectra of the MoS_2_@CC at different precursor concentrations for different reaction times.

X-ray diffraction patterns of MoS_2_@CC electrodes (Fig. 2a–c) confirm the formation of 2H MoS_2_ nanosheets on carbon cloth. The reflections could be indexed to the corresponding lattice planes (002), (004), (100), (102), and (110) of 2H MoS_2_ (JCPDS: 37-1492).

XRD peaks of carbon fiber are also observed in samples while they disappear in sample MC02 deposited for 24 h (Fig. 2c) due to the presence of an increased amount of MoS_2_ nanoflowers. No other impurity peaks are observed in the samples, confirming the growth of pure MoS_2_ nanosheets. From the Raman spectra (Fig. 2d–f), the presence of two prominent vibration modes E_2g_^1^ at around 379 cm^−1^ and A_1*g*_ at around 405 cm^−1^ in all samples indicate in-plane and out-of-plane vibrations of two S atoms in respect of the Mo atom, respectively, further confirming the growth of 2H MoS_2_.^51^ No other impurity peaks are observed in the Raman spectra either.

**Fig. 3:**
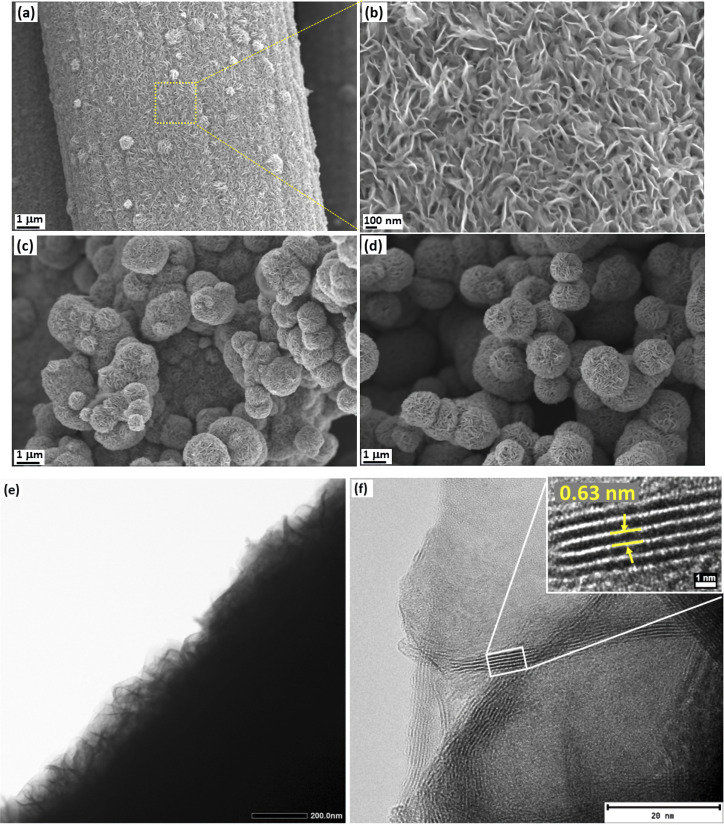
FESEM images of MC02 electrodes at different growth times of (a and b) 6 h, (c) 12 h, and (d) 24 h, and (e and f) TEM images of the MC02@6 h electrodes.

FESEM imaging was used to analyze the coverage and uniformity of MoS_2_ on carbon cloth. The concentrations of the precursors and growth time affect the uniformity and mass loading of MoS_2_ on the surface of the carbon cloth template (Fig. S1†). Therefore, by modifying the precursor concentrations and growth time, it is possible to avoid the agglomeration of MoS_2_ on the carbon cloth while achieving sufficient mass loading. Fig. 3a–d show the FESEM images of the MC02 electrodes. Uniform growth of vertically aligned MoS_2_ nanosheets was observed on the surface in the 6 h experiment MC02 electrodes (Fig. 3a and b). Some spherical nanostructured microscopic features (nanoflowers) are also present on the surface. As the deposition time increases, more MoS_2_ nanoflowers form on the vertically grown MoS_2_ (Fig. 3c and d), leading to their agglomeration and entire population throughout the surface. Therefore, the mass loading of the electrode varied significantly depending on the synthesis parameters, from 5.1 g m^−2^ (MC005@12 h) to 184.6 g m^−2^ (MC02@24 h) on carbon cloth of 132 g m^−2^ specific mass (Table S1†). TEM imaging (Fig. 3e and f) of the MC02 electrode at a growth time of 6 h (MC02@6 h) also confirms uniform growth of the vertically aligned MoS_2_ with an interlayer spacing of 0.63 nm.

**Fig. 4:**
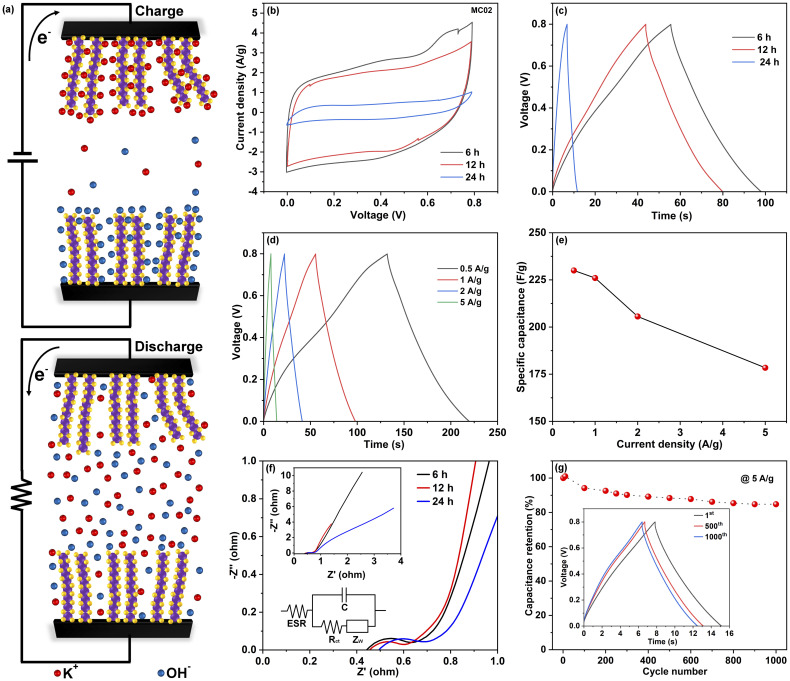
(a) Schematic of the electrochemical system, (b and c) CV at 50 mV s^−1^ and GCD curves at 1 A g^−1^ of the MC02 electrode based SC at different reaction times (note: some error data points are visible in the CV data), (d) GCD curves of the MC02@6 h-based SC at different current densities, (e) variation of specific capacitance *versus* current densities, (f) Nyquist plots of MC02 based SCs with different synthesis times, (g) cycling stability of the MC02@6 h based SCs for 1000 cycles, at a current density of 5 A g^−1^ (inset shows the GCD profiles of the 1^st^, 500^th^ and 1000^th^ cycles for MC02@6 h based SCs, at a current density of 5 A g^−1^).

The electrochemical performance of the electrodes was analyzed using CV and GCD techniques. Fig. 4a illustrates the electrochemical system of the SCs. The CV and GCD curves of MC005 and MC01 electrodes are shown in Fig. S2.† The obtained discharge time is low in MC005 and MC01 devices. Fig. 4b shows the CV curves (acquired at a scan rate of 50 mV s^−1^) of SCs made with the MC02 electrodes. The quasi-rectangular shapes of the CV curves indicate that the devices have both pseudocapacitive (faradaic) and EDLC (non-faradaic) features. In the faradaic process, electrolyte ions (K^+^) are intercalated into the MoS_2_ layers and redox reactions occur (eqn (7)), whereas in the non-faradaic process, the formation of an electric double layer takes place due to the adsorbed ions at the electrode/electrolyte interface (eqn (8)) to facilitate charge storage.^52–54^

7 MoS_2_ + K^+^ + e^−^ ↔ MoS − SK^+^

8
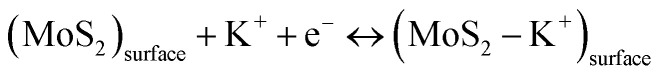


The area of the CV loops of MC02-based SCs decreases with growth time (6 h, 12 h, and 24 h), which implies that the surplus MoS_2_ in the form of nanoflowers does not contribute significantly to the charge storage, which can be explained by their poor electrical contacts with the current collector. These results align well with the GCD curves measured at a current density of 1 A g^−1^ (Fig. 4c), showing a reduction of charge–discharge times for the devices made of MC02 electrodes synthesized at a growth time of 12 and 24 h. The corresponding specific capacitances of the devices were calculated to be 56.5 F g^−1^, 48 F g^−1^, and 6.8 F g^−1^, respectively. (The specific capacitance of respective electrodes is 4 times that of the device, *i.e.*, 226 F g^−1^, 192 F g^−1^, and 27.2 F g^−1^ for MC02 electrodes at growth times of 6 h, 12 h and 24 h electrodes, respectively). Table S1† shows detailed information about mass loading and electrochemical performance of the assembled MoS_2_-based SCs. From these results we can draw a conclusion that generally increasing the precursor concentrations increases the mass loading and the electrode performance whereas increasing the synthesis time increases the mass loading but after a certain point will only add more mass to the electrode without having contribution to charge and energy storage. The electrochemical performance of the MC02@6 h-based SC was further analyzed in detail by systematically varying charge/discharge currents in GCD (Fig. 4d) and voltage scan rates in CV measurements (Fig. S3†). The device exhibits good stability, and no noticeable deviation of the shapes of the CV curves was observed. As displayed in Fig. 4e, the specific capacitances calculated from the GCD curves decreased with increased current densities (230 F g^−1^, 226 F g^−1^, 206 F g^−1^, and 178 F g^−1^ at current densities of 0.5 A g^−1^, 1 A g^−1^, 2 A g^−1^, and 5 A g^−1^, respectively) because of slow ion adsorption and charge transfer at the electrode/electrolyte interface. It is important to note that no obvious voltage drop was observed at the start of discharge even at high specific current densities, indicating the low equivalent series resistance of the device. This makes our device unique among the reported binder-free MoS_2_-based SCs^39^ where high voltage drop was found to limit their practical application. Table 1 lists the electrochemical properties of reported binder free pristine MoS_2_-based SCs on carbon cloth. It is worth noting that while this research is focused on optimizing the synthesis of MoS_2_ on carbon cloth current collectors for supercapacitor electrodes using a straightforward and cost-effective route, capacitances over 500 F g^−1^ have been reported for MoS_2_ based supercapacitors. The performance of MoS_2_ based electrodes can be further improved with tailored aqueous electrolytes^56,57^ as well as with the addition of further metal oxides/sulfides^42,58^ and also by applying conductive polymers with pseudocapacitive properties^59^ reaching capacitances over 3000 F g^−1^.^60^

**Table 1:** Hydrothermally grown binder-free MoS_2_ SC electrodes on carbon cloth

Electrolyte	Specific electrode capacitance (A g^−1^)	Retention/cycles	Ref.
1 M H_2_SO_4_	550.0 F g^−1^ @ 1 A g^−1^	75%/8000	55
PVA-H_2_SO_4_	3.8 F cm^−2^ @ 1 mA cm^−2^	83.3%/10 000	45
0.5 M H_2_SO_4_	170 F g^−1^ @ 1 A g^−1^	—	42
1 M Na_2_SO_4_	151.1 F g^−1^ @ 10 mA cm^−2^	86.1%/2000	41
1 M (NH_4_)_2_SO_4_	1010 F g^−1^ @ 1 A g^−1^	98%/10 000	56
2 M LiCl	1.4 F cm^−2^ @ 9 mV s^−1^	75%/2000	57
6 M KOH	226 F g^−1^ @ 1 A g^−1^	85%/1000	This work

EIS measurements were performed in the frequency range from 100 mHz to 100 kHz, from which the equivalent series resistances (ESR) and charge transfer resistances (*R*_ct_) were assessed according to the Nyquist plot (Fig. 4f). The ESR (total resistance of the current collector, electrolyte, and electrode material) of the MC02@6 h based SC was found to be 0.45 Ω. The diameter of the semicircle on the real axis in the high-frequency region gives a charge transfer resistance of 0.2 Ω, denoting extremely good ion conducting pathways provided by MoS_2_ nanosheets. The Warburg impedance (*Z*_W_), visible as the slope in the low frequency region after the semicircle, generally represents the diffusion of ions within the electrolyte. In SCs based on longer synthesis times the overall resistances are very similar (<1 Ω), with only a small increase of resistances compared to SCs with electrodes having lower MoS_2_ loadings (Fig. S4†). The cycling stability of the MC02@6 h-based SC was assessed using GCD measurements at a current density of 5 A g^−1^ (Fig. 4g). No noticeable voltage drops were found even after 500 or 1000 cycles at the start of the discharge cycles (inset of Fig. 4f), and the capacitance retention was 85% after 1000 charge/discharge cycles.

**Fig. 5:**
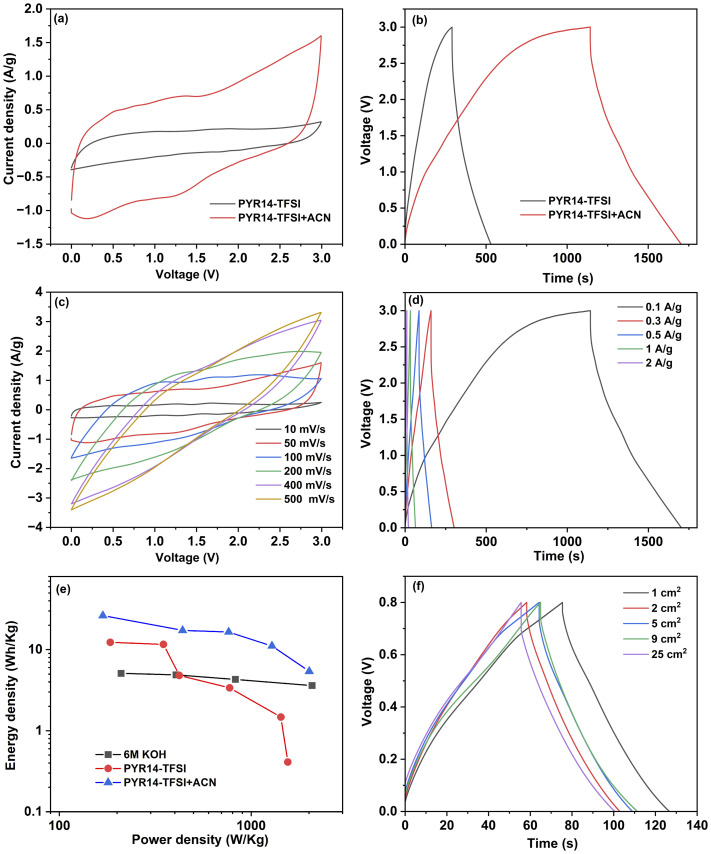
(a) CV curves and (b) GCD curves of the MC02@6 h electrode based SC in IL and the mixture of IL and ACN at 0.1 A g^−1^, (c) CVs at various scan rates (d) GCD at different current densities for the MC02@6 h electrode based SC in the mixture of IL and ACN electrolyte, (e) Ragone plot of MC02@6 h based SCs in aqueous electrolyte and PYR14-TFSI and PYR14-TFSI + ACN ILs and (f) GCD curves of MC02@6 h electrode-based SCs with different electrode sizes ranging from 1 cm^2^ to 25 cm^2^ measured at 1 A g^−1^.

The electrochemical performance of the optimized electrode was further assessed by using an ionic liquid (IL), specifically 1-butyl-1-methylpyrrolidinium bis(trifluoromethanesulfonyl)imide (PYR14-TFSI) as an electrolyte. ILs are considered promising electrolytes for energy storage applications because of their non-volatility, thermal stability, and wide electrochemical window, approximately 3.5 V.^66^ However, ILs exhibit higher viscosity compared to other organic electrolytes, which can reduce their ionic conductivity. Recent research has shown that adding solvents like acetonitrile (ACN) to ILs can decrease their viscosity, thus improving ionic conductivity.^66,67^ The electrochemical performance of Swagelok-type cells assembled with the optimized electrodes (*i.e.* MC02@6 h) was evaluated in both solvent-free PYR14-TFSI IL and a mixture of acetonitrile and PYR14-TFSI in a 1 : 1 mass ratio at a voltage window of 3 V. Fig. 5a shows the CV curves of the MC02@6 h electrode-based SC at a scan rate of 50 mV s^−1^. A CV with an enlarged area was observed for the SC when using a mixture of ACN and PYR14-TFSI electrolyte, in comparison to using PYR14-TFSI IL alone. The CV and GCD curves for the MC02@6 h based SC in PYR14-TFSI are presented in Fig. S5.† The specific capacitance of the SCs was measured from the GCD curves (Fig. 5b) using eqn (1). The specific capacitances of the SC with PYR14-TFSI and the mixture of ACN and PYR14-TFSI were obtained as 10.6 F g^−1^ and 23.8 F g^−1^ at a current density of 0.1 A g^−1^, respectively (the specific capacitances of the electrodes are 42.4 F g^−1^ and 95.2 F g^−1^, respectively). The enhancement in capacitance is primarily attributed to the reduced viscosity of the ILs upon solvent addition, compared to the viscosity of solvent-free ILs. The detailed electrochemical performance of the MC02@6 h-based SC, utilizing a mixture of IL and ACN, was further analyzed. Fig. 5c shows the CV curves of the device at various scan rates ranging from 10 mV s^−1^ to 500 mV s^−1^, while Fig. 5d shows the GCD profiles at different current densities. The SC exhibited specific capacitances of 23.8 F g^−1^, 20.1 F g^−1^, 19.5 F g^−1^, 16.7 F g^−1^, and 12.3 F g^−1^ at current densities of 0.1 A g^−1^, 0.3 A g^−1^, 0.5 A g^−1^, 1 A g^−1^, and 2 A g^−1^, respectively (correspondingly, the specific capacitances calculated for the electrodes themselves were 95.2 F g^−1^, 80.4 F g^−1^, 78 F g^−1^, 66.8 F g^−1^ and 49.2 F g^−1^, respectively) which are similar to the values reported for MoS_2_ electrodes in ILs (Table 2).

**Table 2:** Literature reports on MoS_2_ based electrodes in IL electrolytes

Electrode materials	Electrolyte	Device capacitance	Energy density	Power density	Ref.
Intercalated 1T-MoS_2_	EMIM-BF_4_/MeCN	250 F cm^−3^ (∼32 F g^−1^)	110 W h dm^−3^	1.1 kW dm^−3^	53
Asymmetric MoS_2_/CNTs-MnO_2_	PVDF-HFP/EMIM-BF_4_/EMIM-TFSI	∼50 F g^−1^	124 W h kg^−1^	∼10 kW kg^−1^	61
MoS_3_ and MoS_2_ nanosheets	TEA-BF_4_ +ACN	39.9 F g^−1^	20.7 W h kg^−1^	1.9 kW kg^−1^	62
Micro-holed MoS_2_	EMIM-BF_4_ + ACN	469.5 F cm^−3^	80.5 W h dm^−3^	∼35 kW dm^−3^	63
Exfoliated MoS_2_-graphene	EMIM-BF_4_ + ACN	∼650 F cm^−3^	564.9 W h kg^−1^	6.1 kW kg^−1^	64
MoS_2_-RGO	BMIM-BF_4_	∼54 F g^−1^	0.22 W h dm^−3^	—	65
MoS_2_/CC	PYR14-TFSI	10.6 F g^−1^	12.3 W h kg^−1^	1.5 kW kg^−1^	This work
MoS_2_/CC	PYR14-TFSI + ACN	23.8 F g^−1^	26.3 W h kg^−1^	2.0 kW kg^−1^	This work

Even with the addition of a solvent to ILs, there is an observed increase in voltage drop as the current density rises, with a significant voltage drop of approximately 1.2 V noted at a high current density of 2 A g^−1^, attributed to the slow kinetic properties of the ILs. Fig. 5e shows the comparison of the Ragone plots for the SCs using KOH, PYR14-TFSI, and PYR14-TFSI + ACN as electrolytes. The highest measured energy density and power density of KOH are obtained as 5.1 W h kg^−1^ and 2.1 kW kg^−1^, respectively. The PYR14-TFSI + ACN -based SC shows the highest energy density of 26.3 W h kg^−1^ and power density of 2 kW kg^−1^, respectively, which is higher than that of the solvent-free PYR14-TFSI-based SC. These results indicate that the optimized electrode is also effective with an ionic liquid electrolyte, although it performs better at lower current densities. The lower capacitances measured with ILs compared to KOH can be explained by their larger ions that are unable to intercalate into the MoS_2_ structure. Therefore, without modification of the electrode material, for example by making a MoS_2_–graphene composite,^64^ energy and power densities cannot reach the highest values reported for MoS_2_ based SC electrodes based on aqueous electrolytes.^60^

The feasibility of scaling up the optimized MC02@6 h electrode was assessed by assembling sandwich-type supercapacitors of varying footprint areas from 1 cm^2^ to 25 cm^2^. GCD curves of these devices (Fig. 5f), recorded at a current density of 1 A g^−1^, exhibit very similar profiles, and calculated specific device capacitances of 69 F g^−1^, 63 F g^−1^, 64 F g^−1^, 66 F g^−1^, and 67 F g^−1^ for 1 cm^2^, 2 cm^2^, 5 cm^2^, 9 cm^2^ and 25 cm^2^, respectively. (The corresponding specific capacitances calculated for the electrodes are 276 F g^−1^, 252 F g^−1^, 256 F g^−1^, 264 F g^−1^ and 268 F g^−1^). The negligible deviations of data may be attributed to variations in mass loadings. Accordingly, our experiments suggest that the devices can be effectively scaled up to manufacture robust supercapacitors for use in large-scale applications. It is also worth noting that the mechanical flexibility of the electrodes (Fig. S6†) could potentially enable the manufacturing of bendable supercapacitors similar to those reported earlier with the use of solid-state electrolytes.^41,68–70^

## 4. Conclusions

The present work demonstrated the *in situ* growth of vertically aligned MoS_2_ nanosheets on carbon cloth for high-power supercapacitors using a simple hydrothermal technique. Electrochemical studies infer that the growth time has a significant effect on device performance most likely due to the lack of intimate electrical contacts of MoS_2_ nanoflowers (which populate the collector surface after 12 and 24 h synthesis) with the carbon cloth. Therefore, it is important to find the correct synthesis parameters especially the growth time, as too long growth adds only an inactive mass of material to the electrode. The binder-free MoS_2_ based electrodes grown at a reaction time of 6 h exhibited the highest specific capacitance of 226 F g^−1^ at a current density of 1 A g^−1^. The device showed good stability and capacitance retention of 85% after 1000 charge/discharge cycles. Moreover, the optimized electrode (MC02@6 h) was also effective in an ionic liquid electrolyte providing energy and power densities of 26.3 W h kg^−1^ and 2.0 kW kg^−1^, respectively. The consistent specific capacitance achieved for devices in all scaled-up device sizes indicates the electrode viability for large-scale supercapacitor (SC) applications.

## Data availability

Data for this article, including the figure data, are available at data repository Zenodo at https://doi.org/10.5281/zenodo.12667716.

## Author contributions

Jasna Mannayil: conceptualization, methodology, investigation, visualization, writing – original draft preparation, writing – reviewing and editing. Olli Pitkänen: conceptualization, methodology, visualization, writing – reviewing and editing. Minna Mannerkorpi: methodology, investigation. Krisztian Kordas: conceptualization, supervision, writing – reviewing and editing.

## Conflicts of interest

There are no conflicts to declare.

## Supplementary Material

NA-006-D4NA00368C-s001
