# Supplementary material for: Optimization and scalability assessment of supercapacitor electrodes based on hydrothermally grown MoS2 on carbon cloth
Source: Nanoscale Adv. 2024 Jul 18;6(18):4647–56. doi: 10.1039/d4na00368c (PMC11385806; doi:10.1039/d4na00368c)
Supplement: NA-006-D4NA00368C-s001 [file NA-006-D4NA00368C-s001.pdf]

Electronic Supplementary Material (ESI) for Nanoscale Advances.
This journal is © The Royal Society of Chemistry 2024

Electronic supplementary information

Optimization and scalability assessment of supercapacitor electrodes based on hydrothermally grown MoS_2 on carbon cloth

Jasna Mannayil[1,*], Olli Pitkänen[1,**], Minna Mannerkorpi[2], Krisztian Kordas[1]

[1]Microelectronics Research Unit, University of Oulu, Erkki Koiso-Kanttilan katu 3, 90570, Oulu, Finland

[2]Research Unit of Health Sciences and Technology, University of Oulu, 90220, Oulu, Finland

*Email: jasna.mannayil@oulu.fi
**Email: olli.pitkanen@oulu.fi

Figure S1 shows FESEM images of MC005 and MC01 electrodes, which show uniform MoS2 coverage on MC005 electrodes, while MoS2 nanoflowers aggregated on MC01 electrodes with increasing growth time.

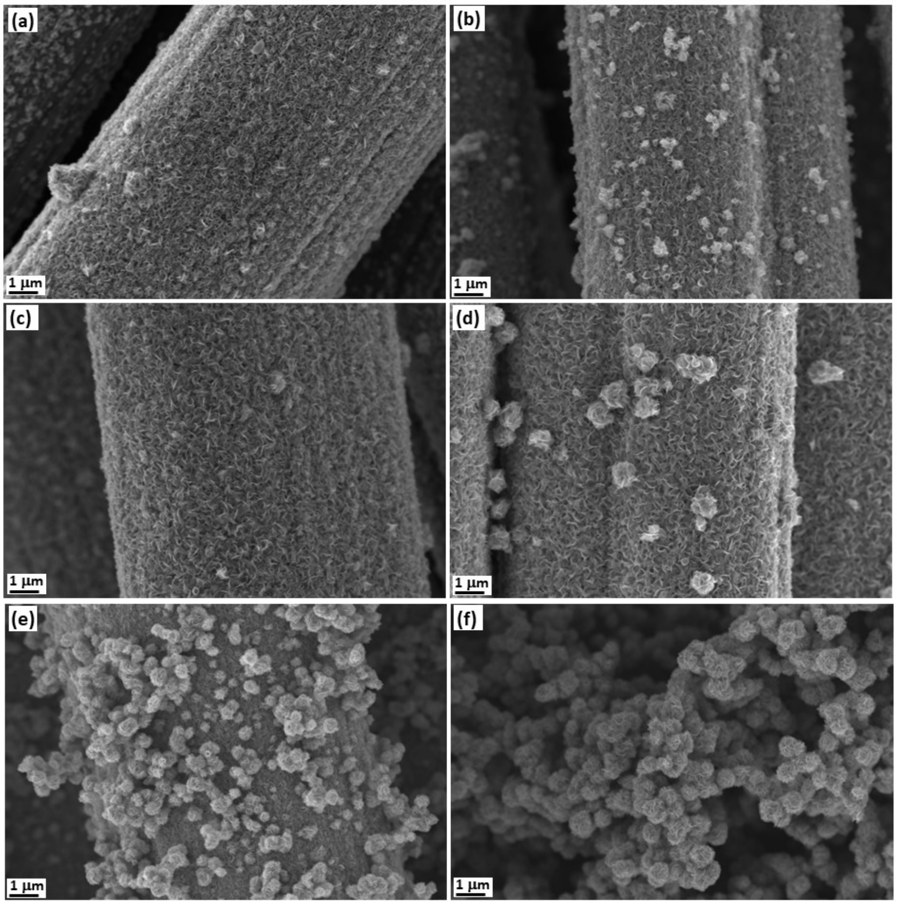

Figure S1. FESEM images of MC005 at different growth times a) 12 h, b) 24 h, c) 36 h, d) 48h, and MC01 at growth time of e) 12 h and f) 24 h.

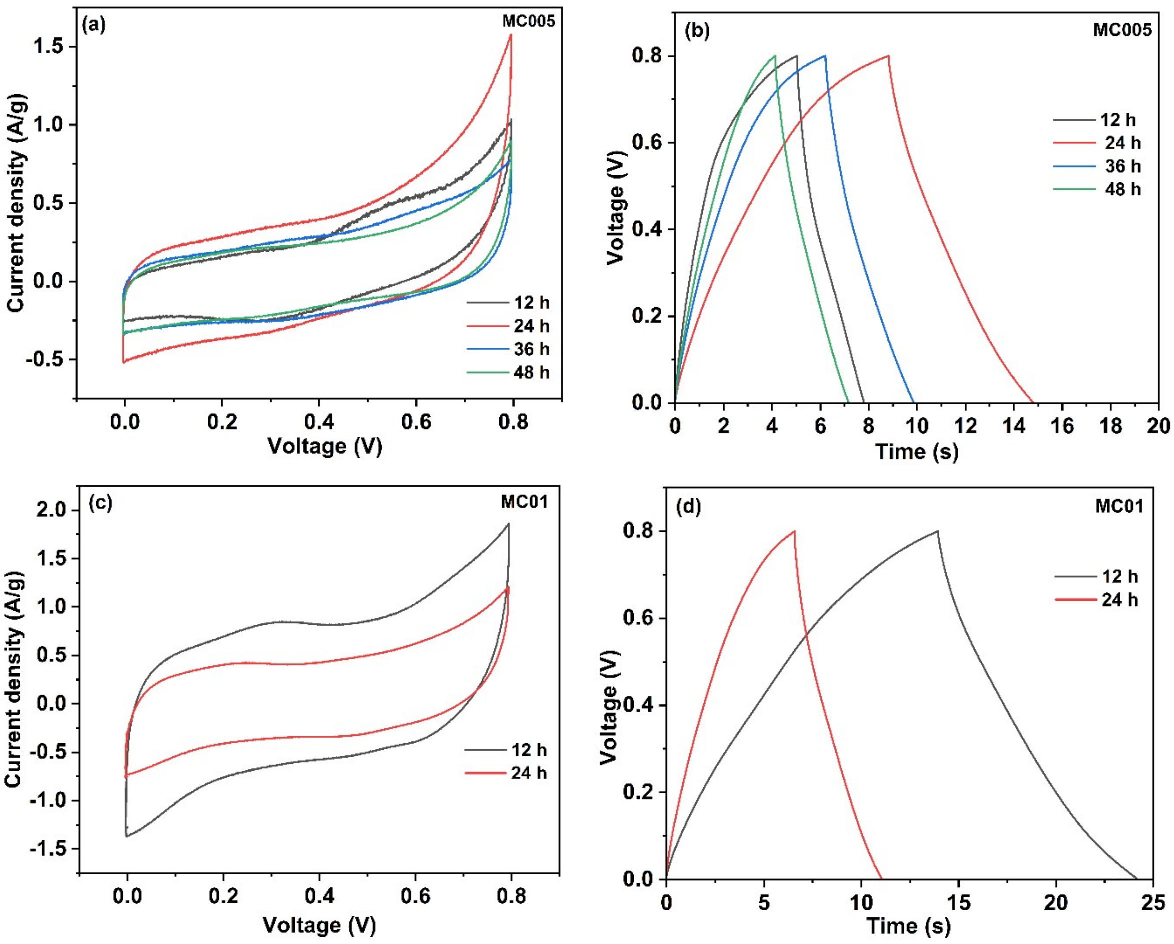

Figure S2. CV curves at a scan rate of 50 mV/s and GCD curves at a current density of 1A/g of (a and b) MC005 and (c and d) MC01 electrodes, respectively, at different growth times.

Table S1. Electrochemical performance of MoS2-based SCs

**Sample name**	**Precursor concentrations (M)**		**Synthesis time (h)**	**Mass loading**		**Specific cap. (F/g)**	
	**Sodium molybdate dihydrate (M)**	**Thiourea (M)**		**Electrode (mg)**	**Electrode area (g/m^2)**	**dev.**	**elec.**
MC005	0.005	0.025	12	0.4-0.5	5.1-6.4	6	24
	0.005	0.025	24	0.8-0.9	10.2-11.5	8.1	32.4
	0.005	0.025	36	0.9-1.1	11.5-14.0	4.9	19.6
	0.005	0.025	48	1.6-1.8	20.4-22.9	4.2	16.8
MC01	0.01	0.05	12	1.8-2.0	22.9-25.5	13	52
	0.01	0.05	24	3.7-3.8	47.1-48.4	17.5	70
MC02	0.02	0.1	6	2.3-2.5	29.3-31.8	56.5	226
	0.02	0.1	12	6.0- 6.2	76.4-78.9	48	192
	0.02	0.1	24	14.3-14.5	182.1-184.6	6.8	27.2

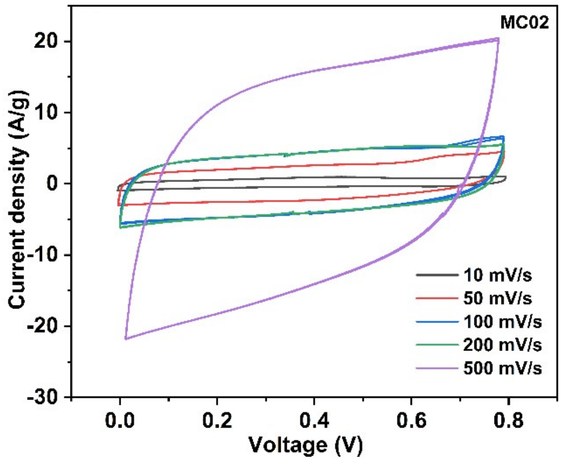

Figure S3. (a) CV curves of MC02@6h-based SCs at different scan rates

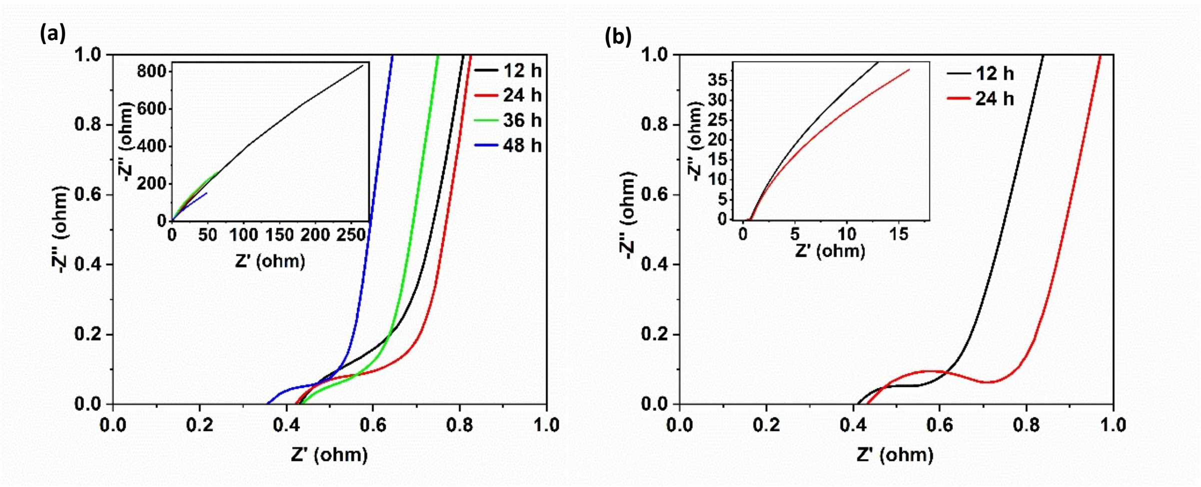

Figure S4. Nyquist plots of (a) MC005 and (b) MC01 based SC with different synthesis times

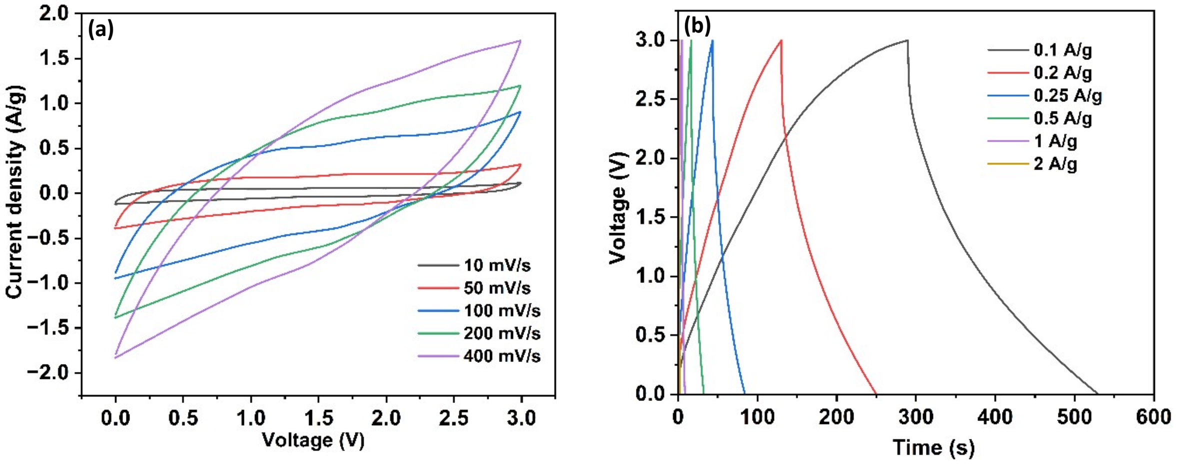

Figure S5. (a) CV at different scan rates and (b) GCD curves at different current densities of MC02@6h SC in PYR14-TFSI IL.

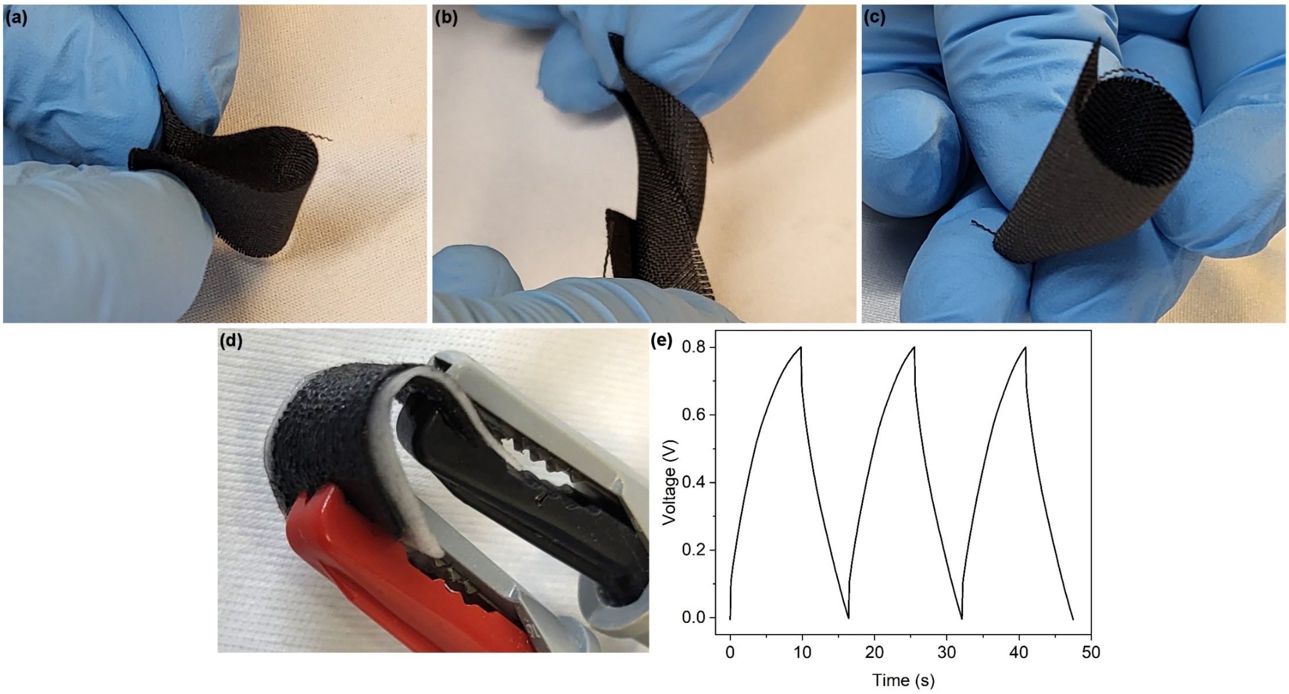

Figure S6. Demonstrations flexibility of the MoS_2@CC electrode material by (a) folding, (b) twisting and (c) rolling. (d) Simplified full cell open assembly of MC02@12h supercapacitor (6M KOH as electrolyte) connected by one sided alligator clips and (e) measured corresponding GCD curves with ~0.3 A/g current density.
